# Arthroscopic vs. open Broström-Gould procedure for chronic lateral ankle instability: a systematic review and meta-analysis

**DOI:** 10.3389/fsurg.2026.1859946

**Published:** 2026-06-18

**Authors:** Jianjun Liu, Jun Wan, Wenjing Zhang, Kai Sun, Ren Zhang

**Affiliations:** 1 Department of Orthopedic Surgery, Jiujiang University Affiliated Hospital, Jiujiang, China; 2 Department of Surgery, Yingtan 184 Hospital, China RongTong Medical Healthcare Group Co. Ltd, Yingtan, China

**Keywords:** arthroscopic, Broström-Gould, chronic lateral ankle instability, meta-analysis, open

## Abstract

**Background:**

Chronic lateral ankle instability (CLAI) is a common complication of ankle sprains, and the Broström-Gould procedure is the preferred option. This study aimed to compare the short-term (with a mean follow-up period of 24 months) clinical efficacy and postoperative recovery rate between arthroscopic and open Broström-Gould surgery in patients with CLAI.

**Methods:**

The present study followed the PRISMA 2020 guidelines. PubMed, Embase, Cochrane Library, Web of Science were searched to 31 December 2025.Meta-analyses were performed using Review Manager (version5.3) for the following outcomes: surgery time, AOFAS scores, K-P scores, VAS scores, Tegner activity scores, Anterior drawer, Talar tilt, Return to sports, Total complication rates, Bias risk evaluation was performed applying the Cochrane Collaboration's Risk of Bias 2 (RoB2) tool and the Newcastle-Ottawa Scale (NOS).

**Results:**

14 studies (875 patients) met criteria, with 421 allocated to open and 454 to arthroscopic groups.There were no statistically significant differences in surgical time [mean difference [MD] = −5.33; 95% confidence interval [CI], −13.68 to 3.01; *p* = 0.21], AOFAS scores (MD = 0.36; 95% CI, −0.23 to 0.96; *p* = 0.23), Tegner activity scores (MD = 0.13; 95% CI, −0.10 to 0.36; *p* = 0.27), anterior drawer test (MD = −0.10; 95% CI, −0.23 to 0.03; *p* = 0.14), talar tilt (MD = −0.00; 95% CI, −0.51 to 0.51; *p* = 0.99) and all complication rates [odds ratio (OR) = 0.88; 95%CI, 0.55 to 1.41; *p* = 0.59]. The arthroscopy group achieved significantly better results in terms of K-P scores (MD = 0.78; 95% CI, 0.02 to 1.54, *p* = 0.04), VAS scores (MD = −0.65; 95% CI, −1.17 to −0.12, *p* = 0.02), and time to return to sports (MD = −3.91; 95% CI, −7.52 to −0.30; *p* = 0.03).

**Conclusions:**

Both techniques show broadly comparable outcomes, with possible earlier return to sport or less pain after arthroscopy, although the certainty of this finding is limited.

**Level of Evidence:**

Level III, systematic review.

This protocol has been registered in the PROSPERO database CRD420261298649.

## Introduction

The ankle joint, a crucial weight-bearing and mobile joint in the human body, is a common site for lower limb sports injuries due to its unique structure (1). An injury to the anterior talofibular ligament (ATFL) is directly associated with ankle sprains (2). While most patients can recover with active conservative treatment, approximately 20% may develop chronic lateral ankle instability (CLAI) due to inadequate conservative management (3). Symptoms such as instability while walking and pain can significantly impact patients’ daily activities and sports performance. Additionally, around 40% of untreated patients may experience long-term complications like talar cartilage injury or osteoarthritis (4). CLAI that fails conservative treatment requires surgical repair of the ligaments to restore normal physiological function.

Currently, the primary surgical approaches in clinical practice involve residual ligament repair and graft reconstruction, with the Broström-Gould procedure emerging as the preferred method for repairing the residual ATFL (5). The traditional Broström-Gould procedure, which entails open surgery to secure the inferior extensor retinaculum and ligament residual end to the fibular insertion site using suture anchors, has been augmented by the emergence of arthroscopy and minimally invasive approaches. The arthroscopic Broström-Gould repair has gained popularity owing to its advantages of minimal trauma, faster recovery, and improved outcomes (6, 7). Nevertheless, debates persist regarding the comparative efficacy in the medium and long term, as well as the speed of postoperative functional recovery between the two techniques (8–10). Although several systematic reviews have compared the two surgical interventions in recent years, the available evidence is constrained by inadequate sample sizes, considerable clinical and methodological heterogeneity among the included studies, and a substantial risk of bias (11–14). Hence, the current systematic review is necessary and well-motivated, and we included 14 studies to conduct this systematic review. The comprehensive analysis aimed to delineate clinical disparities between the two procedures and refine clinical treatment protocols.

## Materials and methods

The present study followed the PRISMA (Preferred Reporting Items for Systematic Meta Analyses) guidelines (15). This study was registered with PROSPERO (CRD420261298649). Two independent investigators (WJ and ZWJ) systematically searched PubMed, Embase, Cochrane Library, Web of Science from inception to December 31, 2025.The search procedure is based on the following keywords: (“ankle instability” OR “chronic lateral ankle instability”) AND (“Broström-Gould” OR “Modified Broström”) AND (“arthroscopy” OR “open surgery”). We first screened the title and abstract of each article to establish eligibility, followed by a full-text assessment of those meeting the criteria. Additionally, we conducted a comprehensive examination of the references cited in the included articles for completeness.

## Inclusion and exclusion criteria

Two researchers (WJ and ZWJ) independently screened eligible studies. Included studies compared arthroscopic vs. open Broström-Gould procedures in patients with MRI-confirmed ATFL disruption, positive anterior drawer test, and ≥6 months of failed conservative treatment, with ≥12 months follow-up, ≥10 patients per group, and English full-text (RCTs, non-RCTs, retrospective cohorts). Excluded were case reports, reviews, animal studies, using suture tape augmentation, concomitant treatment of osteochondral lesions incomplete data, <12 months follow-up, and patients with prior ankle fractures, severe arthritis. Any disagreements were resolved by a third researcher (LJJ).

### Data extraction and quality assessment

Two reviewers (ZWJ and SK) independently extracted pertinent details from the literature, such as the first author, publication date, sample size, patient demographics (age and gender), surgical method, and follow-up duration. Outcome measures encompassed surgery time(minutes),American Orthopedic Foot and Ankle Society (AOFAS) scores, Karlsson–Peterson(K-P) scores, Visual Analogue Scale(VAS) scores, Tegner activity scores, Anterior drawer(mm, stress x-ray), Talar tilt(˚,stress x-ray), Return to sports(weeks), Total complication rates. For studies lacking standard deviations, we contacted authors by email to request the required data. Outcome measures (effect sizes, 95% CI, *I*^2^ values, and corresponding *P* values) were extracted for analysis. Any disagreements were resolved by a third researcher(LJJ). Two researchers (ZWJ and SK) independently evaluated the included literatures; if there were discrepancies, they discussed with a third researcher (LJJ) to re-evaluate the literature quality. For randomized controlled trials, the Cochrane Collaboration's Risk of Bias 2 (RoB2) tool was used to assess the risk of bias (16), classifying each trial into low risk, high risk, or some concerns. Methodological quality of non-randomized controlled studies was evaluated via the Newcastle-Ottawa Scale (NOS) (17). Based on the NOS scoring criteria, studies with scores of 7–9 points were classified as high-quality (low risk of bias), 4–6 points as moderate-quality, and 0–3 points as low-quality.

### Statistical analysis

Data analysis was conducted using Review Manager (RevMan 5.3, Nordic Cochrane Center, Copenhagen, Denmark) for this meta-analysis. Continuous data analysis utilized mean difference (MD) with 95% confidence intervals (95% CI), while dichotomous data analysis employed odds ratios (OR) with 95% CI. Heterogeneity was assessed using the I^2^ test, where I^2^ > 50% and *P* < 0.10 indicated high heterogeneity. In instances of substantial heterogeneity, random-effect models were applied; conversely, fixed-effect models were utilized when heterogeneity was low.

### Publication bias

The funnel plot was used to assess the publication bias in our study.

## Results

### Literature search and screening

**Figure 1:**
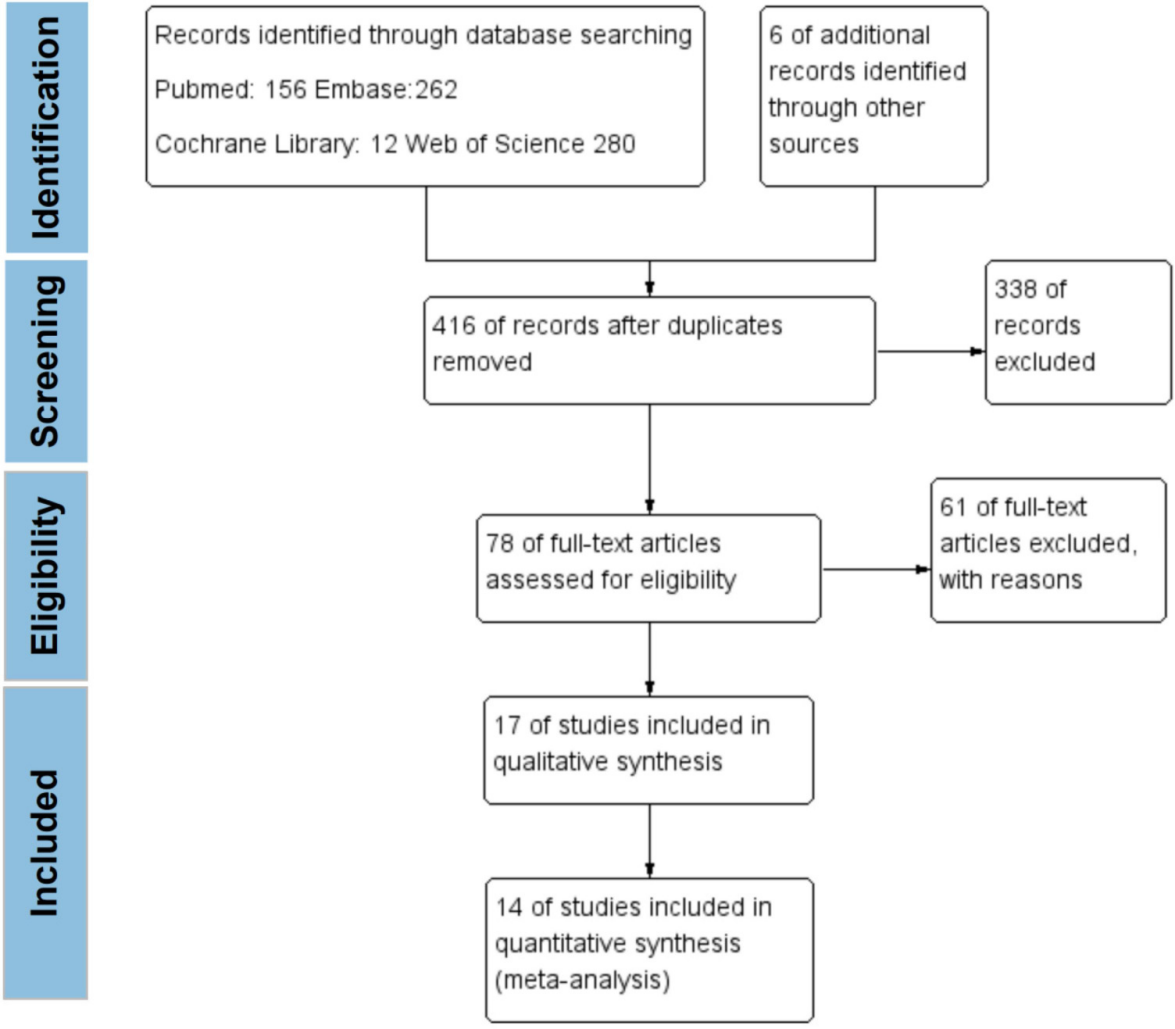
The flow chart of literature screening.

The PRISMA flow diagram (Figure 1) illustrates the literature search and selection process. A total of 716 articles were initially identified, and 14 were included after strict screening (9, 10, 18–29).

### Characteristics of the included studies and risk of bias assessment

**Figure 2:**
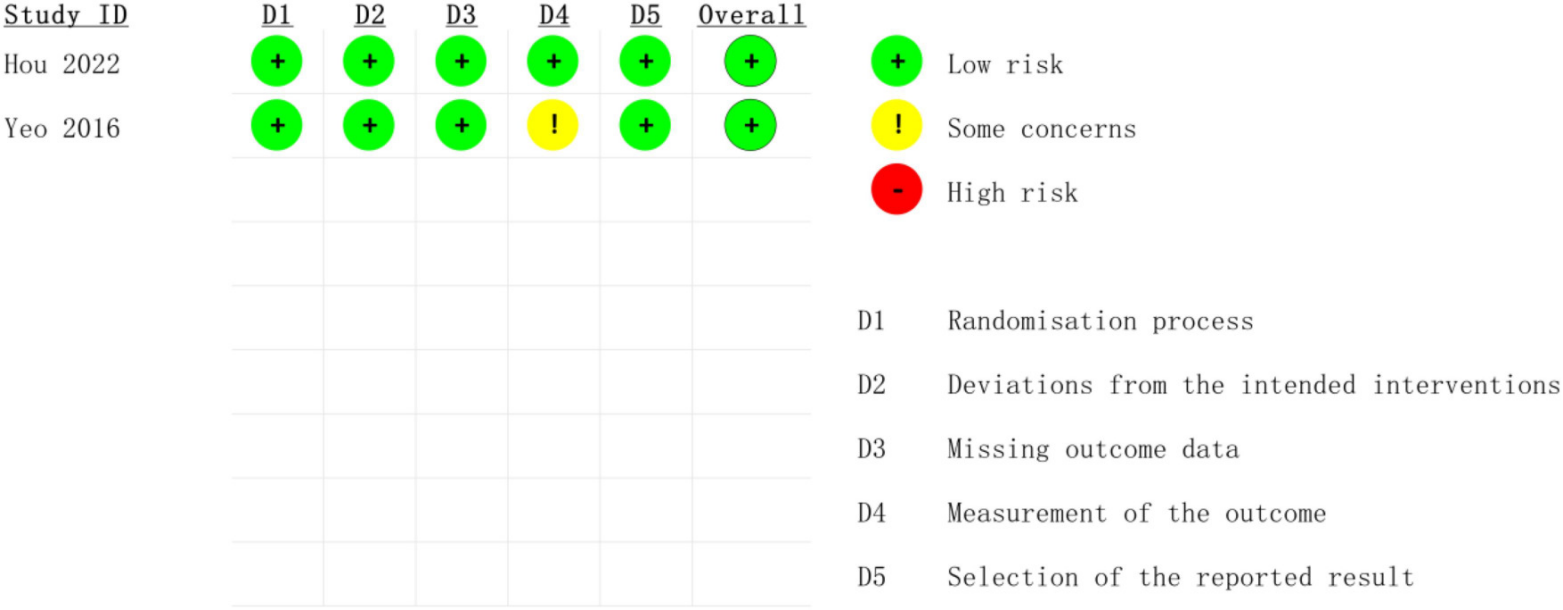
Risk of bias assessment of RCT study.

A total of 14 studies were included, among which 2 were randomized controlled trials (RCTs) and 12 were retrospective cohort studies. The follow-up duration ranged from 12 to 48 months. These studies were conducted in multiple countries, including China, the United States, Canada, the Republic of Korea, and Japan. A total of 875 patients were enrolled, consisting of 421 patients in the open surgery group and 454 patients in the arthroscopy group. The mean age, follow-up duration, and clinical outcomes are presented in Table 1. Two randomized controlled trials (RCTs) were assessed for methodological quality using the RoB 2 tool, with an overall risk of bias rated as low (Figure 2). Twelve retrospective cohort studies were evaluated with the NOS, all of which achieved a score of 7 points or higher, indicating high quality (Table 2).

**Table 1:** Basic characteristics of included studies.

Author/year	Study design/level of evidence	Country	Patients, *n*		Age(years)*		Follow-up (months)*		Clinical outcome
			OB	AB	OB	AB	OB	AB	
Zhao 2025 (18)	RCS/III	China	40	60	31	35	12		①②③④⑤⑨
Yang 2024 (22)	RCS/III	China	28	23	48.9 ± 17.3	46.7 ± 16.7	12		①②④⑨
Wang 2023 (10)	RCS/III	China	50	49	31.92 ± 4.77	31.71 ± 4.99	48		①②③④⑤⑨
Hou 2022 (28)	RCT/I	China	34	36	28.6 ± 4.8	28.3 ± 5.4	24		②③④⑧⑨
Zhou 2021 (19)	RCS/III	China	36	31	31.36 ± 7.79	33.42 ± 6.40	33.06 ± 6.82	29.69 ± 3.40	②③④⑤⑥⑨
Su 2021 (25)	RCS/III	China	40	40	34.27 ± 15.73	38.68 ± 14.23	24		①②③⑥⑦⑨
Xu 2020 (23)	RCS/III	China	35	32	35.8 ± 8.5	33.7 ± 2.3	39.1 ± 9.2	36.5 ± 12.7	②③④⑤⑨
Woo 2020 (24)	RCS/III	Singapore	26	26	31.5 ± 10.3	33.4 ± 10.6	12		②④
Zeng 2019 (20)	RCS/III	China	10	17	27.7 ± 9.7	30.9 ± 6.0	36		①②③⑥⑦⑨
Rigby 2017 (9)	RCS/III	USA	32	30	37.7	47.9	44.4	15.6	②③④⑨⑨
Devries 2019 (29)	RCS/III	USA	12	43	39.5 ± 16	44.7 ± 13.2	21.0 ± 7.1	24.2 ± 7.7	⑧⑨⑨
Li 2017 (27)	RCS/III	China	37	23	28.7 ± 8.7	30.3 ± 10.1	35.5 ± 9.9	39.7 ± 10.3	②③⑤⑨
Yeo 2016 (21)	RCT/I	Korea	23	25	34.3 ± 14.1	35.2 ± 11.8	12		②③④⑥⑦⑨
Matsui 2015 (26)	RCS/III	Japan	18	19	24	48	12		①④⑥⑦⑧⑨

① Surgery time. ② AOFAS. ③ Karlsson-Peterson. ④ VAS. ⑤ Tegner. ⑥ Anterior drawer test. ⑦ Talar tilt. ⑧ Return to sports. ⑨ Complication.

* Data are presented as mean ± s.d. or as mean (range).

OB, Open Broström-Gould; AB, Arthroscopic Broström-Gould; RCS, retrospective cohort study; RCT, randomized controlled trial.

**Table 2:** Quality assessment of the studies by the Newcastle-Ottawa scale.

Study	Selection				Comparability		Outcome			
	Representativeness of the exposed cohort	Selection of external control	Ascertainment of exposure	Outcome of interest was not present at start of study	Main factor	Additional factor	Assessment of outcomes	Sufficient follow-up time	Adequate of follow-up	Total (9/9)
Zhao 2025 (18)	★	★	★	★	★	★	★	★	★	9/9
Yang 2024 (22)	★	★	★	★	★	★		★	★	8/9
Wang 2023 (10)	★	★	★	★	★	★	★	★	★	9/9
Zhou 2021 (19)	★	★	★	★	★	★		★	★	8/9
Su 2021 (25)	★	★	★	★	★	★		★	★	8/9
Xu 2020 (23)	★	★	★	★	★	★		★	★	8/9
Woo 2020 (24)	★	★	★	★	★	★		★	★	8/9
Zeng 2019 (20)	★	★	★	★	★	★		★	★	8/9
Rigby 2017 (9)	★	★	★	★	★	★		★	★	8/9
Devries 2019 (29)	★	★	★	★	★	★		★		7/9
Li 2017 (27)	★	★	★	★	★	★		★	★	8/9
Matsui 2015 (26)	★	★	★	★	★	★		★	★	8/9

★Signifies that the criterion is satisfied, with one point awarded.

### Surgical outcomes

#### Surgery time (minutes)

**Figure 3:**
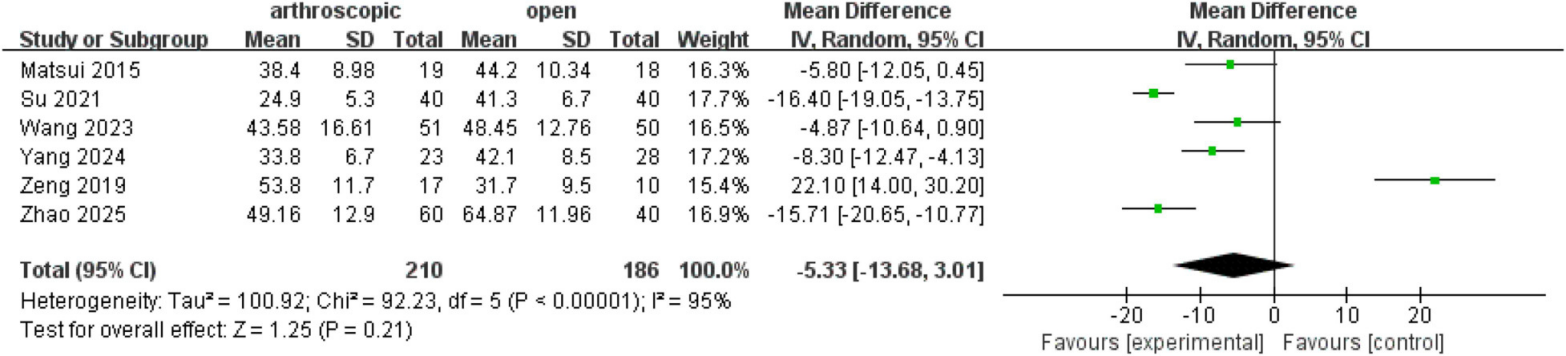
Forest plot, surgery time (minutes).

A total of 6 clinical studies (10, 18, 20, 22, 25, 26) were included to compare the differences in surgical time between arthroscopic and open Broström-Gould procedures for the treatment of CLAI. Results of a meta-analysis using a random-effects model demonstrated no statistically significant difference in surgical time between the two groups (MD = −5.33; 95% CI, −13.68 to 3.01; *P* = 0.21).Substantial heterogeneity was observed among the included studies (*I*^2^ = 95%, *P* < 0.001), indicating significant variations in surgical technical details, surgeon experience, and comorbidities of the included patients (Figure 3).

### AOFAS scores

**Figure 4:**
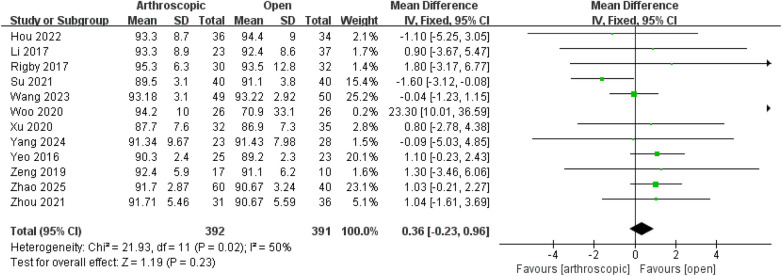
Forest plot, AOFAS scores.

A total of 12 studies (9, 10, 18–25, 27, 28) were initially included for the meta-analysis of postoperative AOFAS scores. Using a fixed-effects model, no significant difference in postoperative AOFAS scores was observed between arthroscopic and open Broström-Gould procedures (MD = 0.36; 95% CI, −0.23 to 0.96; *P* = 0.23). After excluding the primary source of heterogeneity[Woo 2020 (24)]11 studies were retained for sensitivity analysis. Heterogeneity was substantially reduced after exclusion (*I*^2^ = 4%, *P* = 0.40), indicating high consistency across the remaining studies (Figure 4).

### K-P scores

**Figure 5:**
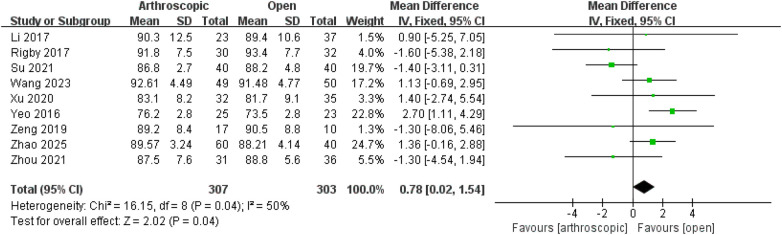
Forest plot, K-P scores.

A meta-analysis comparing *K-P* scores in arthroscopic versus open Broström-Gould repair revealed a significantly higher score in the arthroscopic group (MD = 0.78; 95% CI, 0.02 to 1.54, *P* = 0.04) based on 9 studies (9, 10, 18–21, 23, 25, 27) with moderate heterogeneity (*I*^2^ = 50%, *P* = 0.04).Sensitivity analysis, excluding two studies [Su 2021 (25) and Yeo 201 6^21^], eradicated heterogeneity (*I*^2^ = 0%, *P* = 0.64) but rendered the effect non-significant (MD = 0.76; 95% CI, −0.23 to 1.76, *P* = 0.13). These results imply that the initial significant difference was influenced by the excluded studies, suggesting a lack of statistical robustness in the findings. (Figure 5).

### VAS scores

**Figure 6:**
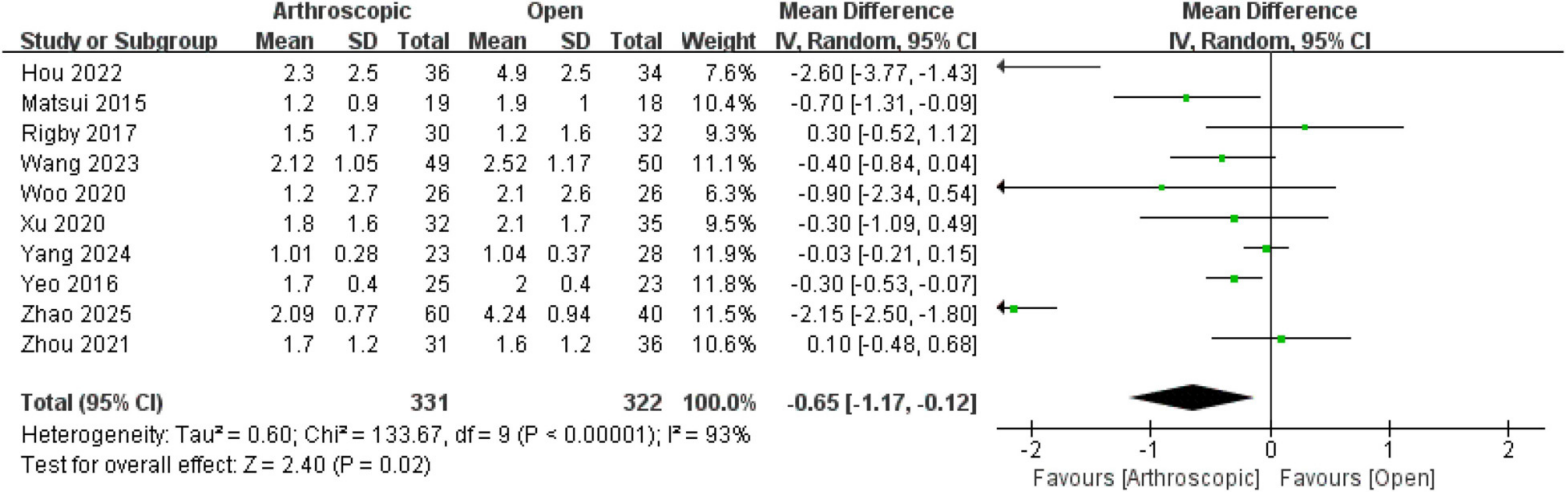
Forest plot, VAS scores.

A meta-analysis of VAS pain scores from 10 studies (9, 10, 18, 19, 21–24, 26, 28) indicated that arthroscopic repair yielded significantly lower pain scores compared to open repair (MD = −0.65; 95% CI, −1.17 to −0.12, *P* = 0.02). Given the high heterogeneity (*I*^2^ = 93%), we performed a sensitivity analysis. After excluding two studies [Hou 2022 (28), Zhao 2025 (18)], heterogeneity decreased to 35%, Although arthroscopic surgery still shows certain statistical advantages (MD = −0.17; 95% CI, −0.29 to −0.04, *P* = 0.008), the difference is minimal and thus has limited clinical guiding significance (Figure 6).

#### Tegner activity scores

**Figure 7:**
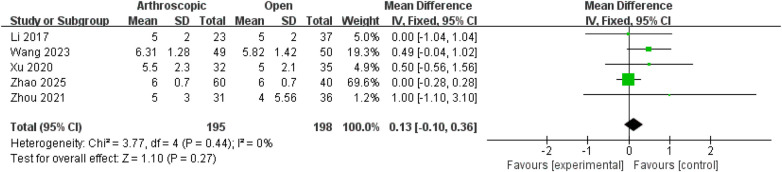
Forest plot, tegner scores.

A total of 5 studies (10, 18, 19, 23, 27) were included in this meta-analysis to compare the effects of arthroscopic and open surgeries on the postoperative Tegner activity scores. Heterogeneity among the included studies was extremely low (*I*^2^ = 0%, *P* = 0.44). Pooled analysis using a fixed-effects model revealed no statistically significant difference in postoperative Tegner activity scores between the arthroscopic and open surgery groups (MD = 0.13; 95% CI, −0.10 to 0.36; *P* = 0.27) (Figure 7). This finding indicated a high consistency in the efficacy of the two surgical approaches regarding postoperative recovery of motor function.

### Anterior drawer(mm)

**Figure 8:**
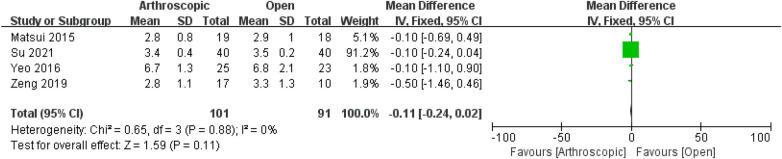
Forest plot, anterior drawer(mm).

A total of 4 studies (20, 21, 25, 26) were included in this meta-analysis to compare the outcomes of the anterior drawer test after arthroscopic and open surgeries for CLAI. There was extremely low heterogeneity among the included studies (*I*^2^ = 0%, *P* = 1.00). Pooled analysis with a fixed-effects model demonstrated no statistically significant difference in the postoperative numerical values of the anterior drawer test between the arthroscopic and open surgery groups (MD = −0.10; 95% CI, −0.23 to 0.03; *P* = 0.14) (Figure 8). This finding based on objective measurements indicated a high consistency between the two surgical approaches in restoring the anatomical stability of the ankle joint.

**Figure 9:**
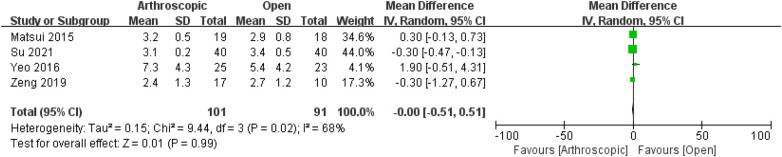
Forest plot, talar tilt(˚).

### Talar tilt(˚)

A total of 4 studies (20, 21, 25, 26) were included in this meta-analysis to compare the talar varus angle between arthroscopic and open Broström-Gould procedures for the treatment of ankle instability. The forest plot revealed moderate heterogeneity among the studies (*I*^2^ = 68%, *P* = 0.02), so a random-effects model was applied for the pooled analysis. The pooled mean difference (MD = −0.00; 95% CI, −0.51 to 0.51; *P* = 0.99) (Figure 9). Current evidence suggests that arthroscopic and open Broström-Gould procedures yield equivalent effects in improving the talar varus angle in patients with ankle instability, with no significant differences observed between the two techniques. The observed heterogeneity may stem from the methods used to measure talar varus angle also differed between studies, with some employing weight-bearing radiographs and others using non-weight-bearing imaging, which could introduce measurement bias and contribute to the observed heterogeneity.

### Return to sports (weeks)

**Figure 10:**
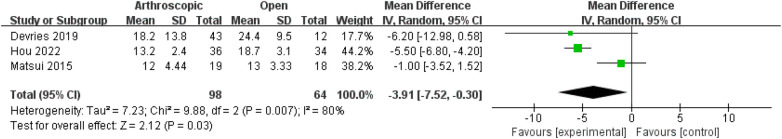
Forest plot, return to sports(weeks).

A total of 3 studies (26, 28, 29) were included in this meta-analysis to compare the time to return to sports (in weeks) between arthroscopic and open surgeries. Owing to significant statistical heterogeneity among the included studies (*I*^2^ = 80%, *P* = 0.007), a random-effects model was used for pooled analysis. The results showed that the mean time to return to sports in the arthroscopic group was significantly shorter than that in the open surgery group, with a pooled mean difference (MD = −3.91; 95% CI, −7.52 to −0.30; *P* = 0.03) (Figure 10). The high heterogeneity (*I*^2^ = 80%) among the studies was mainly attributed to differences in the athletic levels of included patients (elite athletes vs. recreational participants) and variations in postoperative rehabilitation protocols. Due to the small number of included studies and high heterogeneity, this outcome as low-certainty evidence.

### Total complication

**Figure 11:**
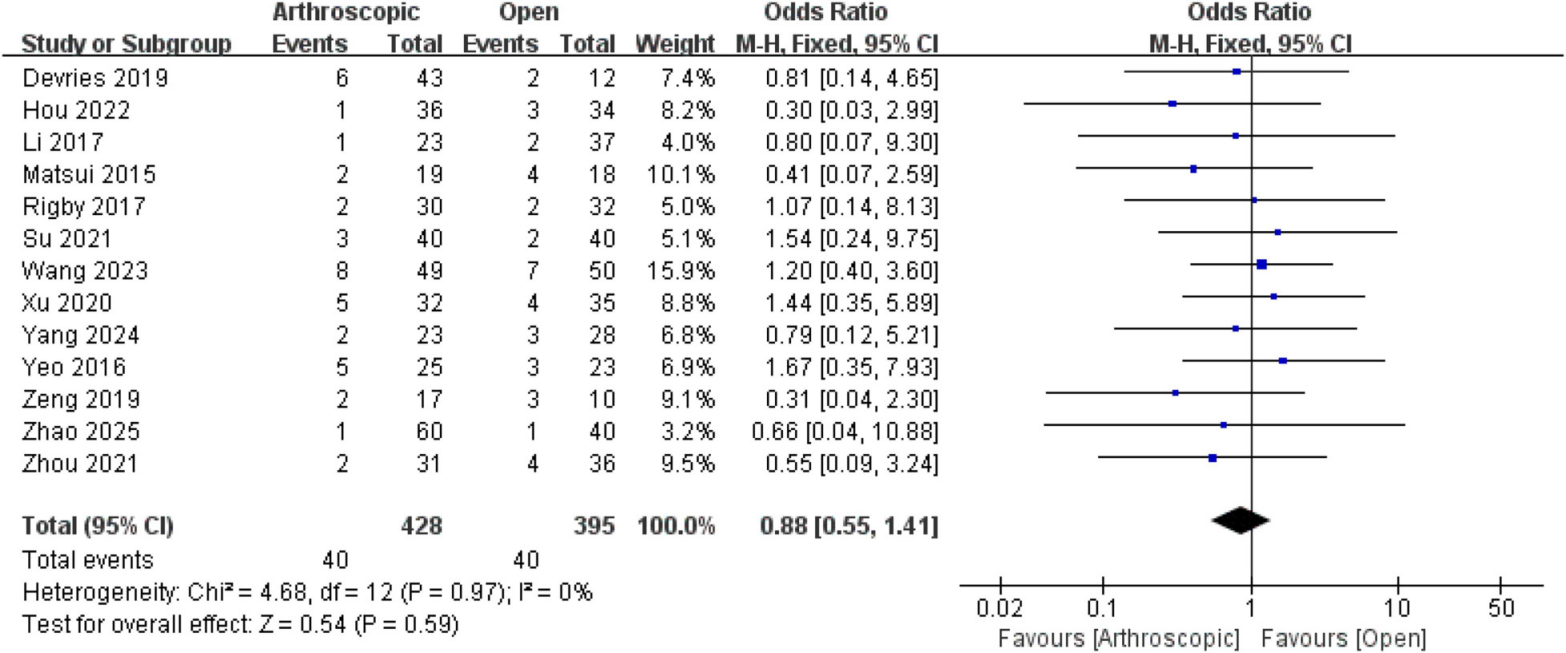
Forest plot, total complication.

A total of 13 studies (9, 10, 18–23, 25–29) were included in this meta-analysis to compare the risk of postoperative complications between arthroscopic and open surgery for ankle instability. Pooled analysis showed no statistically significant heterogeneity across the included studies (*I*^2^ = 0%, *P* = 0.97), and a fixed-effects model was therefore used for data pooling. The results indicated no significant difference in the incidence of postoperative complications between the arthroscopic group and the open surgery group (OR=0.88; 95% CI, 0.55 to 1.41; *P* = 0.59) (Figure 11). These findings confirm a high level of consistency in postoperative safety between the two surgical approaches.

## Publication bias

**Figure 12:**
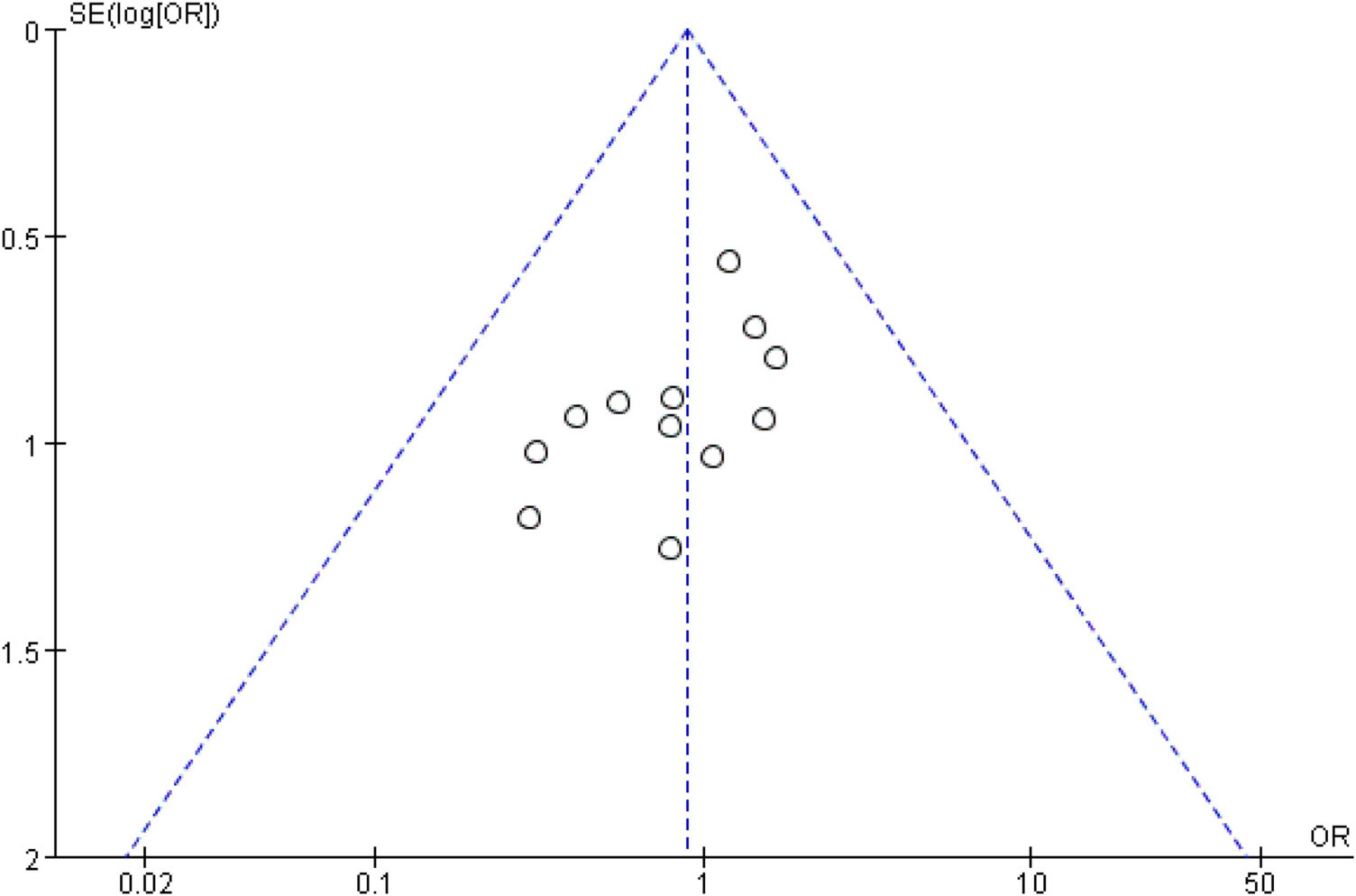
Funnel plot for total complication.

Publication bias was evaluated using funnel plots for total complication (Figure 12), The distribution of scatter spots was symmetrical on both sides of the funnel plots, suggesting no evidence of publication bias.

## Discussion

Currently, many studies have reported that the repair of ATFL via either open or arthroscopic Broström-Gould procedure is safe, feasible, and yields favorable outcomes (8–10, 19, 27). This meta-analysis extensively compared the clinical effectiveness and safety of arthroscopic versus open Broström-Gould repair for lateral ankle instability to offer evidence-based recommendations for clinical practice. In line with prior systematic reviews (13, 14), our results validate the widespread acceptance of arthroscopic Broström-Gould repair, mainly due to its benefits of less trauma, quicker recovery, and enhanced functional outcomes. The arthroscopic approach, in contrast to the open method, circumvents extensive soft tissue dissection, thus decreasing intraoperative trauma, reducing postoperative pain, and expediting the recovery process, aligning with the characteristics of minimally invasive surgery.

In the analysis of surgery time included in this study, 6 retrospective studies were enrolled. In the single-study analysis, most studies (5/6) indicated a shorter surgery time in the arthroscopic group, but the difference was statistically significant in only some of them; only 1 study showed a significantly shorter operative time in the open group. This difference may be attributed to the fact that arthroscopy allows direct visualization during bone tunnel drilling, which reduces fluoroscopy time and consequently shortens the operative time. As reported by Zhao et al. (18), a learning curve exists for both surgical techniques, but arthroscopic surgery requires more cases to achieve proficiency. Go et al. (30) also noted in their study that due to the need for precise identification of anatomical structures and proficient manipulation of surgical instruments, the experience of surgeons typically influences the duration of arthroscopic surgery. The learning curve demonstrates that as operative proficiency improves, surgical time can be reduced by 40%.This finding further confirms the influence of procedural details and surgeon proficiency on clinical variables.

The ankle joint function was analyzed using the AOFAS scores, K-P scores, Tegner activity scores and Return to sports. The results of this Meta-analysis showed that no statistically significant difference between arthroscopic and open surgery in improving the postoperative AOFAS scores. In comparative studies (10, 22, 25, 28, 29) of AOFAS scores evaluated at various time points, early arthroscopic surgery demonstrates initial benefits within this scoring framework, yet these advantages wane over time. This trend may be attributed to the minimally invasive nature of arthroscopic surgery, resulting in reduced tissue trauma and facilitating prompt mobilization. Regarding the K-P scores, although the heterogeneity between studies was eliminated through sensitivity analysis, the pooled effect size was only 0.76 points, which was also far below the threshold of the recognized minimal clinically important difference (MCID) (31). This suggests that despite the more robust results after excluding heterogeneous studies, the actual differences between the two groups in postoperative ankle joint stability, functional recovery, and patients’ subjective feelings were negligible. Unlike the AOFAS and K-P scores, the Tegner activity scores focuses on the long-term recovery of patients’ athletic ability. This study included 5 high-quality studies, and the difference in the Tegner activity scores between arthroscopic surgery and open surgery was only 0.13 points, with no statistical significance. There are relatively few clinical comparative studies on return to sports after surgery. Hou et al. (28) conducted the first study comparing postural control and muscle function between arthroscopic and open surgeries. The results showed that arthroscopic ligament repair allowed patients to return to sports more quickly than open surgery. However, this was only observed in the follow-up results at 6 months, with no significant differences seen in longer-term follow-up.

Clinically, stress radiography such as the anterior drawer test and talar tilt is commonly used to evaluate postoperative recovery of ankle laxity. A talar displacement exceeding 3 mm in the anterior drawer test or a talar inversion angle > 10°indicates persistent ankle instability (32). The anterior drawer test is the objective gold standard for evaluating ankle reduction and stability (33). The results of the present study showed that the difference in postoperative anterior talar translation between arthroscopic and open surgery group was only −0.11 points, with no statistically significant difference (*P* = 0.11). The present meta-analysis demonstrated that arthroscopic and open Broström-Gould procedures resulted in equivalent postoperative talar tilt angles in patients with ankle instability. This finding carries significant clinical implications: although arthroscopic surgery offers the advantages of being minimally invasive and associated with faster rehabilitation, it is not superior to open surgery in restoring the anatomical stability of the ankle joint. This is inconsistent with the conclusions of some single studies claiming “superior stability with arthroscopy” suggesting that the reported advantages in previous single-center studies may be influenced by surgeon experience or patient selection bias (13). The present large-sample pooled evidence further supports the equivalence of the two procedures in terms of anatomical outcomes.

Postoperative complications serve as a core objective indicator for evaluating surgical safety. Guelfi et al. (34) reported that the overall complication rate of arthroscopic repair of lateral ligaments was 0%–35%, compared with 0–29.5% for open repair. Across the 13 included studies, both open and arthroscopic surgical approaches for ankle instability demonstrated low overall postoperative complication rates(open 10.1%;arthroscopic 9.3%). Among them, the incidence of superficial peroneal nerve(SPN) injury is relatively high in arthroscopic surgery, while the incidence of superficial incision infection is relatively high in open surgery (7, 13, 35), with occasional variations in knot pain and joint instability across individual studies. However, the pooled analysis revealed no statistically significant differences in the incidence of any of the four complication types between the two surgical groups, indicating comparable safety profiles of open and arthroscopic interventions for this condition (Table 3). Of course, there are other complications with lower incidences, such as deep vein thrombosis, secondary distal fibular fracture, and pseudoaneurysm. We believe that with the continuous improvement of surgical techniques, these complications will be gradually reduced.

**Table 3:** Details of complication among two groups.

Author/year	Joint instability,%		SPN* injury,%		Knot pain,%		Wound infection,%	
	OB	AB	OB	AB	OB	AB	OB	AB
Hou 2022 (28)	5.9 (2/34)	2.8 (1/36)	0.0 (0/34)	0.0 (0/36)	0.0 (0/34)	0.0 (0/36)	0.0 (0/34)	0.0 (0/36)
Zhou 2021 (19)	0.0 (0/36)	0.0 (0/31)	2.8 (1/36)	3.2 (1/31)	2.8 (1/36)	0.0 (0/31)	0.0 (0/36)	0.0 (0/31)
Su 2021 (25)	0.0 (0/40)	0.0 (0/40)	5.0 (2/40)	5.0 (2/40)	0.0 (0/40)	2.5 (1/40)	0.0 (0/40)	0.0 (0/40)
Xu 2020 (23)	0.0 (0/35)	0.0 (0/32)	5.7 (2/35)	9.4 (3/32)	0.0 (0/35)	6.3 (2/32)	5.7 (2/35)	0.0 (0/32)
Zeng 2019 (20)	0.0 (0/10)	0.0 (0/17)	0.0 (0/10)	5.9 (1/17)	10.0 (1/10)	0.0 (0/17)	20 (2/10)	5.9 (1/17)
Rigby 2017 (19)	0.0 (0/32)	0.0 (0/30)	6.3 (2/32)	3.3 (1/30)	0.0 (0/32)	0.0 (0/30)	0.0 (0/32)	0.0 (0/30)
Devries 2019 (29)	0.0 (0/12)	0.0 (0/43)	8.3 (1/12)	2.3 (1/43)	0.0 (0/12)	0.0 (0/43)	8.3 (1/12)	2.3 (1/43)
Li 2017 (27)	0.0 (0/37)	0.0 (0/23)	0.0 (0/37)	0.0 (0/23)	0.0 (0/37)	0.0 (0/23)	0.0 (0/37)	0.0 (0/23)
Yeo 2016 (21)	0.0 (0/23)	4.0 (1/25)	8.7 (2/23)	12.0 (3/25)	0.0 (0/23)	8.0 (2/25)	4.3 (1/23)	0.0 (0/25)
Matsui 2015 (26)	0.0 (0/18)	0.0 (0/19)	5.6 (1/18)	10.5 (2/19)	0.0 (0/18)	0.0 (0/19)	16.7 (3/18)	0.0 (0/19)
Yang 2024 (22)	0.0 (0/28)	0.0 (0/23)	0.0 (0/28)	4.3 (1/23)	0.0 (0/28)	0.0 (0/23)	3.5 (1/28)	0.0 (0/23)
Wang 2023 (10)	0.0 (0/50)	0.0 (0/49)	4.0 (2/50)	12.2 (6/49)	0.0 (0/50)	0.0 (0/49)	10.0 (5/50)	4.1 (2/49)
Zhao 2025 (18)	0.0 (0/40)	0.0 (0/60)	2.5 (1/40)	1.7 (1/60)	0.0(0/40)	0.0(0/60)	0.0(0/40)	0.0(0/60)

* SPN, superfical peroneal nerve.

With the advancement of arthroscopic techniques, some researchers advocate utilizing double-anchor or knotless anchor repair to enhance the repair of the ATFL. Meta-analyses (36) have revealed no significant discrepancies in AOFAS scores and Karlsson Ankle Function Score(KAFS) between single-anchor and double-anchor repairs. In recent years, knotless anchor fixation has gained favor, with studies demonstrating its superiority over knotted anchor fixation in terms of functional outcomes and complication rates (37). Controversy still exists regarding whether the calcaneofibular ligament (CFL) requires concomitant repair in the setting of chronic ankle instability. Based on a cadaveric study by Lee et al. (38) anatomical reconstruction of the ATFL alone could provide adequate ankle stability, and additional CFL repair was therefore deemed unnecessary.

This study has several limitations. First, only two randomized controlled trials were included, while the majority of the remaining studies were retrospective. Therefore, more high-quality randomized controlled trials with rigorous methodologies are required to validate the reliability of the present findings. Second, clinical heterogeneity in surgical technique, anchor constructs, augmentation, and concomitant procedures, the overall conclusions should still be interpreted with caution. Third, due to inconsistent rehabilitation protocols and variable definitions of return to sport across included studies, performance bias and measurement bias were relatively prominent. Finally, further large-scale, well-designed randomized controlled trials with standardized surgical procedures and consistent outcome definitions are warranted to verify and update the current evidence.

## Conclusion

This meta-analysis confirms arthroscopic and open Broström-Gould procedures yield equivalent clinical efficacy and safety for CLAI. Arthroscopy is associated with less postoperative pain and potentially earlier return to sports, thus representing a valuable minimally invasive alternative. However, the numerical difference between the two is far below the MCID, so it should be interpreted with caution in clinical practice. Further studies with larger sample sizes and randomized controlled trials (RCTs) are needed to validate this conclusion.
